# Implementation of Multigene Germline and Parallel Somatic Genetic Testing in Epithelial Ovarian Cancer: SIGNPOST Study

**DOI:** 10.3390/cancers13174344

**Published:** 2021-08-27

**Authors:** Dhivya Chandrasekaran, Monika Sobocan, Oleg Blyuss, Rowan E. Miller, Olivia Evans, Shanthini M. Crusz, Tina Mills-Baldock, Li Sun, Rory F. L. Hammond, Faiza Gaba, Lucy A. Jenkins, Munaza Ahmed, Ajith Kumar, Arjun Jeyarajah, Alexandra C. Lawrence, Elly Brockbank, Saurabh Phadnis, Mary Quigley, Fatima El Khouly, Rekha Wuntakal, Asma Faruqi, Giorgia Trevisan, Laura Casey, George J. Burghel, Helene Schlecht, Michael Bulman, Philip Smith, Naomi L. Bowers, Rosa Legood, Michelle Lockley, Andrew Wallace, Naveena Singh, D. Gareth Evans, Ranjit Manchanda

**Affiliations:** 1 Wolfson Institute of Population Health, Barts CRUK Cancer Centre, Queen Mary University of London, Charterhouse Square, London EC1M 6BQ, UK; d.chandrasekaran@qmul.ac.uk (D.C.); m.sobocan@qmul.ac.uk (M.S.); olivia.evans@nhs.scot (O.E.); li.sun1@lshtm.ac.uk (L.S.); f.gaba@qmul.ac.uk (F.G.); 2 Department of Gynaecological Oncology, Barts Health NHS Trust, London EC1 1BB, UK; arjun.jeyarajah@nhs.net (A.J.); alexandra.lawrence5@nhs.net (A.C.L.); elly.brockbank@nhs.net (E.B.); s.phadnis@nhs.net (S.P.); 3 Divison for Gynaecology and Perinatology, University Medical Centre Maribor, 2000 Maribor, Slovenia; 4 School of Physics, Engineering and Computer Science, University of Hertfordshire, Hatfield AL10 9AB, UK; o.blyuss@qmul.ac.uk; 5 Department of Paediatrics and Paediatric Infectious Diseases, Sechenov First Moscow State Medical University, Moscow 119991, Russia; 6 World-Class Research Center “Digital Biodesign and Personalized Healthcare”, Sechenov First Moscow State Medical University, Moscow 119991, Russia; 7 Department of Medical Oncology, Barts Health NHS Trust, London EC1A 7BE, UK; rowan.miller2@nhs.net (R.E.M.); shanthini.crusz@nhs.net (S.M.C.); 8 Department of Medical Oncology, Barking, Havering & Redbridge University Hospitals, Essex RM7 0AG, UK; tinamills-baldock@nhs.net (T.M.-B.); mary.quigley5@nhs.net (M.Q.); fatima.el-khouly@nhs.net (F.E.K.); 9 Department of Health Services Research, Faculty of Public Health & Policy, London School of Hygiene & Tropical Medicine, London WC1H 9SH, UK; rosa.legood@lshtm.ac.uk; 10 Department of Pathology, Barts Health NHS Trust, London E1 1FR, UK; r.f.l.hammond@smd14.qmul.ac.uk (R.F.L.H.); asma.faruqi@nhs.net (A.F.); giorgia.trevisan1@nhs.net (G.T.); laura.casey5@nhs.net (L.C.); naveenasingh7@gmail.com (N.S.); 11 North East Thames Regional Genetics Service, Great Ormond Street Hospital, London WC1N 3JH, UK; lucy.jenkins@gosh.nhs.uk (L.A.J.); munaza.ahmed@gosh.nhs.uk (M.A.); ajith.kumar@gosh.nhs.uk (A.K.); 12 Department of Gynaecology, Barking, Havering & Redbridge University Hospitals, Essex RM7 0AG, UK; r.wuntakal@nhs.net; 13 Manchester Centre for Genomic Medicine, Saint Marys Hospital, Manchester M13 9WL, UK; george.burghel@mft.nhs.uk (G.J.B.); helene.schlecht@mft.nhs.uk (H.S.); michael.bulman@mft.nhs.uk (M.B.); philip.smith@mft.nhs.uk (P.S.); Naomi.Bowers@mft.nhs.uk (N.L.B.); andrew.wallace@mft.nhs.uk (A.W.); Gareth.Evans@mft.nhs.uk (D.G.E.); 14 Barts Cancer Institute, Queen Mary University of London, Charterhouse Square, London EC1M 6BQ, UK; m.lockley@qmul.ac.uk

**Keywords:** ovarian cancer, *BRCA*, genetic testing, germline, somatic, *RAD51C*, *RAD51D*, *BRIP1*

## Abstract

**Simple Summary:**

Multigene testing in ovarian cancer has received increased support due to its‘ applicability for cancer treatment and the impact it has on cancer prevention in families. This study shows that multi-gene germline and somatic testing uptake after counselling by a member of the multidisciplinary cancer clinical team in women with ovarian cancer, was high (97%). A total of 15.5% of women were identified to have germline *BRCA1/BRCA2* pathogenic variants and 7.8% had somatic *BRCA1/BRCA2* pathogenic variants. A total of 2.3% patients had *RAD51C/RAD51D/BRIP1* pathogenic variants. We found that 11% of germline pathogenic variants were large-genomic-rearrangements and were missed by somatic testing. Our findings support prospective parallel somatic-&-germline panel testing to maximize variant identification.

**Abstract:**

We present findings of a cancer multidisciplinary-team (MDT) coordinated mainstreaming pathway of unselected 5-panel germline *BRCA1/BRCA2/RAD51C/RAD51D/BRIP1* and parallel somatic *BRCA1/BRCA2* testing in all women with epithelial-OC and highlight the discordance between germline and somatic testing strategies across two cancer centres. Patients were counselled and consented by a cancer MDT member. The uptake of parallel multi-gene germline and somatic testing was 97.7%. Counselling by clinical-nurse-specialist more frequently needed >1 consultation (53.6% (30/56)) compared to a medical (15.0% (21/137)) or surgical oncologist (15.3% (17/110)) (*p* < 0.001). The median age was 54 (IQR = 51–62) years in germline pathogenic-variant (PV) versus 61 (IQR = 51–71) in *BRCA* wild-type (*p* = 0.001). There was no significant difference in distribution of PVs by ethnicity, stage, surgery timing or resection status. A total of 15.5% germline and 7.8% somatic *BRCA1/BRCA2* PVs were identified. A total of 2.3% patients had *RAD51C/RAD51D/BRIP1* PVs. A total of 11% germline PVs were large-genomic-rearrangements and missed by somatic testing. A total of 20% germline PVs are missed by somatic first *BRCA*-testing approach and 55.6% germline PVs missed by family history ascertainment. The somatic testing failure rate is higher (23%) for patients undergoing diagnostic biopsies. Our findings favour a prospective parallel somatic and germline panel testing approach as a clinically efficient strategy to maximise variant identification. UK Genomics test-directory criteria should be expanded to include a panel of OC genes.

## 1. Introduction

Ovarian cancer (OC) is the leading cause of deaths from gynaecological cancers, with 240,000 new cases and 152,000 deaths occurring worldwide annually [1]. GLOBOCAN data suggest the number of cases from OC will increase by 26% in the UK and 47% worldwide, respectively, over the next 20 years [1]. Standard treatment approaches have been associated with limited long-term OC survival of ~30% [2]. However, the progress over the last 10–15 years has provided the foundations for a precision medicine [3] approach for OC management, involving inherited cancer susceptibility genes. *BRCA1/BRCA2* pathogenic and likely pathogenic variants (henceforth termed ‘pathogenic variants’ or ‘PVs’) account for most of the known inheritable risk of OC. Around 11–18% of OC have germline *BRCA1/BRCA2* PV and another 6–9% have a somatic *BRCA1/BRCA2* PV in the tumour tissue alone which is not inherited. Women with germline *BRCA1/BRCA2* PVs have a cumulative risk by age 80 of 17–44% for developing EOC and 69–72% for developing breast cancer (BC) [4].

Genetic testing for OC susceptibility genes has recently received an impetus through increasing applicability for cancer treatment and eligibility for clinical trials. The proteins coded by *BRCA1/BRCA2* are essential in the homologous recombination repair (HRR) of double stranded DNA breaks, whilst PARP (poly ADP ribose polymerase) is an essential component of single-strand DNA repair. Inhibition of PARP increases double strand breaks and prevents HRR deficient (HRD) tumour cells from surviving chemotherapy induced DNA damage, leading to synthetic lethality [5]. Germline as well as somatic *BRCA* mutated OC have been shown to benefit from ‘PARP inhibitor’ (PARP-i) therapy with improved progression free survival at both recurrent and more recently primary settings [5,6,7,8,9]. Therefore, knowledge of *BRCA* status at the time of diagnosis has become pivotal in the guidance of treatment options. Genetic testing for germline *BRCA1/BRCA2* PVs in EOC was commissioned by NHS-England in 2015, [10] and has been recommended by other published guidelines over the last few years [11]. More recently, the American Society of Clinical Oncology (ASCO) [12], the British Gynaecological Cancer Society (BGCS) [13] and the European Society of Medical Oncology (ESMO) [14] have advocated for somatic testing too.

However, HRD can arise through somatic and germline PV in a wide range of OC susceptibility genes [15]. Approximately 50% of high-grade serous OC are characterised by HRD suggesting additional mechanisms other than *BRCA* mutations play a significant role [14]. HRD assays are now available and are beginning to be used in clinical practice [14]. Further moderate risk OC susceptibility genes in the HRR pathway, such as, *RAD51C, RAD51D* and *BRIP1* with lifetime OC-risks of 5.8 to 13% have been identified and their risks validated [16,17]. Testing for additional genes of clinical utility [18] can lead to wider therapeutic benefit. ASCO now recommends germline *BRCA* testing within the context of a multigene panel [12]. In addition to targeted therapy, identification of PVs offers opportunities for cancer surveillance and prevention for secondary cancers in index patients as well as cascade testing in relatives. Unaffected relatives with PVs can access relevant surgical prevention and screening options which have well established clinical benefit. This includes risk-reducing salpingo-oophorectomy (RRSO) to reduce their OC risk [19,20]; MRI/mammography screening, or risk reducing mastectomy (RRM) [21], or chemoprevention with selective oestrogen receptor modulators (SERM) to reduce their BC risk [22].

Over recent years, many models of care delivery for OC genetic testing have been implemented into clinical practice [23,24,25]. There has been great variation in these clinical pathways, with strategies varying with respect to (a) whom to test (unselected or restricted by histology such as for high-grade serous OC or restricted by age, such as under 70 years); (b) what to test (either germline only, or somatic only, or both) and (c) in which order to test (parallel or sequential); (d) which genes to test (*BRCA* only or multiple genes); and (e) who provides counselling and testing (genetics teams in genetics clinics, genetics professional embedded in oncology clinics, medical oncologists, surgical oncologists, or clinical nurse specialists (CNS)). Despite guidelines, historically, the overall uptake and access to genetic testing across health systems has remained poor, with only 20–30% eligible patients accessing testing [26,27]. Obstacles to introducing routine somatic testing at diagnosis have been attributed to reasons like cost, access/availability of validated somatic testing in a National Health Service (NHS) accredited laboratory and additional resources required to process tumour samples [28]. Most studies to date report clinical experience of implementing *BRCA* testing. Reports of systematic prospective parallel germline panel and somatic genetic testing are limited. We present our experience and findings of implementing a cancer multidisciplinary team (MDT) coordinated mainstreaming pathway of unselected 5-panel germline *BRCA1, BRCA2, RAD51C, RAD51D, BRIP1* and parallel somatic *BRCA1/BRCA2* testing in all women with high grade non-mucinous epithelial OC in the Systematic Genetic Testing for Personalised Ovarian Cancer Therapy (SIGNPOST) study (ISRCTN: 16988857) in women from North East London Cancer Network (NELCN). We report on the somatic testing success rates with different types of sample ascertainment. Moreover, importantly we highlight the discordance between germline and somatic testing strategies incorporating testing data from NELCN as well as the Manchester NHS Foundation trust.

## 2. Materials and Methods

### 2.1. Pre-Test Counselling and Recruitment

Women ≥ 18 years with high-grade non-mucinous epithelial OC, who were newly diagnosed or under follow-up in the NELCN, were offered parallel germline testing for *BRCA1, BRCA2, RAD51C, RAD51D, BRIP1* genes and concomitant *BRCA1/BRCA2* somatic genetic testing. This was undertaken through the SIGNPOST study (ISRCTN: 16988857). Newly diagnosed patients were identified from gynaecological oncology MDT meetings and consented for genetic testing during their primary treatment. Patients undergoing surveillance post-treatment, were identified through follow-up surgical and medical oncology clinics as well as pathology and clinical databases. Eligibility for genetic testing was established by the treating clinician. Patients received written pre-test education information regarding the advantages, disadvantages and implications of genetic-testing. Pre-test genetic counselling and consent was undertaken at routine clinic visits. This was led initially by medical and surgical oncology consultants, and subsequently also undertaken by cancer CNSs. Psychological support was offered by CNSs within the cancer services.

### 2.2. Germline and Somatic Testing

Testing was undertaken by clinically accredited NHS laboratories. A 4 mL EDTA blood sample was taken for germline genetic testing for *BRCA1, BRCA2, RAD51C, RAD51D* and *BRIP1*. Germline testing for NELCN samples was undertaken for *BRCA1, BRCA2, RAD51C, RAD51D* and *BRIP1* at the North East Thames Regional Genomics Laboratory (Great Ormond Street Hospital), while for Manchester samples testing for *BRCA1* and *BRCA2* was undertaken at the Genomic Diagnostic Laboratory at the North West Genomic Laboratory Hub. This was carried out using next generation sequencing (NGS; Agilent SureSelect and Illumina NextSeq) of the coding region, sequenced to a minimum depth of 30 reads, including intron/exon splice boundaries. Sanger sequencing was also carried out to confirm variants detected during the NGS screen. Additionally, exon deletions/duplications in *BRCA1* and *BRCA2* genes were detected using Exome Depth. Multiplex ligation-dependent probe amplification (MLPA; MRC Holland) kits P002-D1 and P090-C1, respectively.

Somatic testing was undertaken using formalin fixed paraffin embedded (FFPE) tissue specimen from diagnostic biopsies, or up front cytoreductive surgery or post-chemotherapy cytoreductive surgery as appropriate. FFPE blocks were reviewed by a consultant histopathologist to identify areas with >20% tumour content and therefore deemed suitable for somatic testing. The specimens were processed and sent as either 5 × 5 µM thick unstained sections, or as 3 mm core biopsies from paraffin blocks. Unstained slides were preferred for small volume diagnostic biopsies and in <20% neoplastic content. Tumour blocks were selected by the pathologist and graded as <20%, 20–50% and >50% neoplastic content. Testing was undertaken in two NHS accredited diagnostic laboratories. Majority NELCN and Manchester samples were analysed at the Manchester Genomics Laboratory while a few NELCN samples were also tested at the Royal Marsden Hospital laboratory. Detection of variants is dependent on the percentage of tumour infiltration, DNA input concentration and DNA quality. DNA extracted from FFPE tissue was analysed in the coding regions of *BRCA1* and *BRCA2*, using NGS and minimum variant allele depth was 10×. The analysis was performed with Molecular Diagnostics Information Management System v-4.0, based on genome hg19 or GeneRead DNAseq v2 Human Breast Cancer Panel (Qiagen) and Illumina NGS. Mutation and variant calling by custom bioinformatic analysis pipeline validated to detect SNVs and small insertion/deletion mutations ( <40 bp) to 5% mutant allele frequency (MAF).

Variants were classified using the ACGS and CanVIG guidance in force (https://www.acgs.uk.com/quality/best-practice-guidelines/ (accessed on 5 January 2021)) [29,30]. Common, high frequency benign and likely benign variants were filtered bioinformatically from a curated list of variants whilst all other variants were assessed by a registered Clinical Scientist. In case of discordance between the germline and somatic samples, a further repeat analysis was undertaken and second report issued. Reports from both germline and somatic tests were sent to the referring clinician for disclosure to the patients.

Validation of 3 mm FFPE punch biopsies for high-volume somatic testing:

Somatic testing using NGS on FFPE specimens has been validated on 5 × 5µM thick unstained sections. [31] In order to minimise delay without compromising DNA yield, particularly for archival FFPE tissue, 3 mm punch biopsies from FFPE tumour blocks were validated for diagnostic somatic testing. Following review by a gynaecological oncology histopathologist, a 5 mm area with high tumour content (>20%) was marked on the Haematoxylin and Eosin (H&E) stain slide. Keyes punch biopsy (routinely used for skin biopsy) was used to core out 3 mm sample from corresponding area in FFPE block. Five 5µM thick unstained sections were also cut from same block. Five matched 3 mm cores and unstained sections were compared for DNA yield.

### 2.3. Test Result Management

Most patients including all those diagnosed with a PV were given their test result and counselled in an outpatient clinic by their consenting and treating cancer clinician. A small proportion of patients on long-term follow up declined an additional hospital visit and were given the result by post. All patients with a PV were referred to North East Thames regional genetics service team for additional post-test genetic counselling and facilitating predictive testing in family members.

We report on testing undertaken between 01/05/2017 to 31/12/2019 across the NELCN, which provides cancer care to a ~1.7 M population covering six NHS hospitals. Patient demographic and clinical data were extracted from electronic patient records, and FH questionnaires completed by the patient. Positive (or strong) FH was defined as any index case of high-grade non-mucinous epithelial OC and breast cancer or epithelial OC in a first-degree or second-degree relative. Patients who had previously undergone genetic testing as they had been referred to clinical genetics in view of a strong FH, were excluded from mainstreaming, but are included in the analysis of prevalence estimates. For the analysis of discordance between germline and somatic *BRCA1/BRCA2* testing we also include data of 116 unselected OC cases from Manchester NHS Foundation trust who underwent parallel germline and somatic testing. The testing procedures and offer of testing was similarly undertaken in Manchester but germline testing was restricted to *BRCA1* and *BRCA2*.

Descriptive statistics were used for baseline characteristics. PV and wild type groups were compared for ethnicity, age, FH, histology, stage, timing of surgery, chemotherapy response score, and residual disease status. Variables associated with number of pre-test consultations (1 or >1) were explored for type of clinician undertaking counselling, disease status at time of counselling (new diagnosis or on follow up) and treatment status (whether undergoing active treatment or not).

Wilcoxon rank-sum test and Fisher’s exact or Chi-square tests were used to test the difference in means and proportions correspondingly. Two-sided *p*-values were reported for all statistical tests. Statistical analysis was undertaken in R version 3.5.1 and SPSS version 26.

## 3. Results

### Pathway Development

Development of the genetic testing pathway was preceded by a wide consultation with the regional clinical geneticists, genetic counsellors, surgical and medical oncologists, CNS, clinical scientists from genetic laboratories, patient representatives and *BRCA* charity leads. Patient representatives and charity leads expressed a preference for genetic testing to be provided at diagnosis, to be made available all patients including those remained under surveillance post-treatment, and for provision for adequate pre-test counselling and informed consent.

In preparation of a cancer MDT coordinated mainstreaming genetic testing service, all gynaecological cancer MDT members (surgical oncologists, medical oncologist, pathologist and CNS) attended small group teaching sessions led by the regional lead in clinical genetics and a gynaecological oncologist with a long-standing special interest and significant experience in cancer genetics, counselling and testing. This covered principles of Mendelian inheritance, OC susceptibility genes and associated cancer risks; the principles, structure and factors specific to genetic counselling; as well as the developed local testing and referral pathways. Knowledge questionnaires were completed by attendees to ensure appropriate understanding of issues. Following pathway implementation, ongoing professional support for the cancer MDT team was provided by gynaecological cancer precision prevention service, with support from the regional clinical genetics team. Pre-counselling written information was developed in collaboration with the major stakeholders and provided to all patients. Additionally, service management meetings across the broader group with representation from medical and surgical oncologist, lead clinical geneticist, clinical scientists from genetic laboratories, lead histopathologist were held every 6–9 months.

Counselling, Recruitment and Genetic Testing:

A total of 310 patients with high-grade non-mucinous epithelial OC who were eligible for genetic testing were identified across the NELCN. This included 188 newly diagnosed women and 122 patients on follow up post-treatment. Of these women seven were excluded: four died prior to commencing treatment, one was unable to consent due to dementia and learning difficulties and two declined genetic testing. The remainder 303 untested patients remained eligible for testing and received pre-test genetic counselling. Of these patients 7/122 (6%) under surveillance had previously undergone germline *BRCA1/BRCA2* mutation testing through clinical genetics due to a strong FH of BC or OC fulfilling prior standard clinical criteria for genetic testing. They were offered and underwent extended panel testing for *RAD51C, RAD51D* and *BRIP1* along-with somatic testing. Overall, we found a 97.7% uptake of parallel multi-gene germline and somatic testing via the cancer MDT mediated mainstreaming pathway.

All of the patients were counselled and consented by a member of the cancer MDT, with 45% (*n* = 137) by a medical oncology member, 36% (*n* = 110) by a surgical oncology member and 18% (*n* = 56) by a CNS. The majority required a single pre-test consultation (78%) prior to consenting, whereas 18% (*n* = 54) required two consultations, 4% (*n* = 13) required three and one patient required four consultations prior to decision to undergo testing (Table 1). The number of pre-test counselling sessions needed varied significantly depending on the clinical professional undertaking counselling. Counselling by CNS was more frequently associated with needing more than one consultation (53.6% (30/56)) compared to counselling by a medical oncologist (15.0% (21/137)) or a surgical gynae-oncologist (15% (17/110)) (*p* < 0.001). The number of consultations required did not significantly differ whether (a) the patient was newly diagnosed or under follow up; and (b) if they were undergoing active treatment or not (Table 1).

Patient demographics and clinical characteristics are summarised in Table 2. The median age at OC diagnosis was 54 years (IQR 51–62) in germline PV compared with 61 (IQR 51–71) in *BRCA* wild type (*BRCA*-WT) (*p* = 0.001) patients. In germline *BRCA1/BRCA2/RAD51C/RAD51D/BRIP1* PVs, 44.4% (24/54) had a positive FH compared to 11.3% (28/249) of sporadic tumours (*p* < 0.001) (Table 2). Thus 55.6% of PVs would have been missed by using FH alone. Only 2/7 of *RAD51C/RAD51D/BRIP1* PVs had a positive FH. Ethnicity of OC cases included 196 (64.7%) White, 28 (9.2%) Black, 52 (17.2%) South Asian and 27 (8.9%) were classed as ‘other’. In women with somatic *BRCA1/BRCA2* PV, the median age at diagnosis was 61 (IQR 59–66) and 13% (2/15) had a positive FH. Most PVs had a high-grade serous (HGS) histology except one *BRCA1* with grade 3 endometrioid carcinoma and one *BRIP1* with mixed epithelial adenocarcinoma. There was no significant difference in distribution of PVs by ethnicity, stage at diagnosis, timing of surgery or resection status (Table 2). In post-chemotherapy cytoreductive surgery specimens, chemotherapy response score (CRS) of 3 (minimal residual disease) was recorded in 13/69 (18.8%) germline and somatic PVs compared to 13/234 (5.6%) of *BRCA*-WT tumours (*p* = 0.025).

Validation of 3 mm FFPE punch biopsies for somatic testing:

Analysis of 3 mm Keyes punch biopsy and 5 × 5µM unstained sections from the same FFPE tumour block demonstrated comparable DNA concentration and yield; therefore, archived tumour samples of patients under follow-up were processed as 3 mm core which proved time-efficient, as it reduced consultant pathologist time needed for review, retrieval and marking of slides. This is therefore likely to be more cost-efficient (Table 3).

Tumour testing results were available for 232 NELCN cases. Of the 71 cases without tumour testing results, 40 cases lacked available archived tumour tissue for analysis (unable to retrieve from pathology archive or surgery at another cancer centre); and 25 archived cases lacked any tissue with adequate neoplastic content (minimal diagnostic biopsy or post-chemotherapy tumour necrosis leaving no viable sample for analysis); and six test results were awaited at the time of analysis (delays due to COVID pandemic). Of these 71 cases without a somatic result, nine had a PV on germline genetic testing (four *BRCA1*, three *BRCA2*, one *RAD51C*, one *RAD51D*). Of the 232 NELCN tumour samples that underwent testing, 19 (8.9%) failed analysis due to fragmented DNA or low neoplastic content. Of these failed 19 cases, one had a *BRCA1* PV and one a *RAD51D* PV on germline testing. Further details on tumour tissue processing are provided in Table 4. The failure rate was higher for diagnostic biopsies (22.9%; 11/48) compared to primary cytoreductive surgical specimens (5.4%; 6/110) and post-chemotherapy surgical specimens (2.7%; 2/74). Primary-surgery specimens that failed analysis were due to fragmented DNA. There were 11 (out of 232) samples categorised with <20% neoplastic content, of which five (45%) were subsequently found to be adequate for analysis (Table 4). A majority of the samples were sent for analysis as 3 mm core biopsies from paraffin blocks (174/232, 75%) and the rest as unstained slides (58/232, 25%). Failure rates were 3/174 (1.7%) in 3 mm cores and 16/58 (27.6%) in unstained slides, respectively. However, 6/16 failed analysis in the unstained slides group had <20% neoplastic content. In our centre, tissue was preferentially sent as unstained slides if neoplastic content was <20% or the sample was a small volume diagnostic biopsy.

Genetic testing results:

Following multi-gene germline testing, 54 germline PVs were identified in 303 women from the NELCN cohort (Supplementary Table S1). Of these PVs, 33 (11%) were *BRCA1*; 14 (4.6%) *BRCA2*, 2 (0.7%) *RAD51C*, 3 (1.0%) *RAD51D* and 2 (0.7%) *BRIP1*). Six PVs were large genomic rearrangements (LGR) and detected by MLPA: four in *BRCA1*, one in *BRCA2* and one in *RAD51C*. The germline VUS rate in *BRCA1/BRCA2* was 3.3% (*n* = 10) and 3.3% (*n* = 10) in *RAD51C/RAD51D* and *BRIP1* (Table 5). Germline *BRCA1/BRCA2* testing in the Manchester cases identified 11 (9.5%) PVs, of which 8 (6.9%) were *BRCA1* and 3 (2.6%) were *BRCA2* PVs (Supplementary Table S1). Additionally, one *BRCA1* VUS was identified. The median age of the Manchester cohort was 63 years (IQR = 55–72). Overall, 14 Manchester patients had a strong FH of cancer. Four of the eleven germline PV had a strong FH, while seven lacked a strong FH and would have been missed without unselected testing. Combining data from NELCN and Manchester series, the total *BRCA1/BRCA2* germline PV rate was 15.5% (65/419) and *BRCA1/BRCA2* germline VUS rate was 2.6% (11/419).

A total of 232 tumour *BRCA1/BRCA2* results were available at the time of analysis from NELCN cases. Somatic *BRCA1/BRCA2* PVs were detected in 15 (6.6%) cases and the VUS rate was 2.2% (*n* = 5). Tumour *BRCA1/BRCA2* testing in 116 Manchester cases identified 7 (6%) *BRCA1* and 5 (4.3%) *BRCA2* somatic PVs as well as 1 (0.9%) *BRCA1* and 1 (0.9%) *BRCA2* somatic VUS each (Table 5). The total *BRCA1/BRCA2* somatic PV rate was 7.8% (27/348) and somatic VUS rate was 2% (7/348). A germline or somatic PV was identified in 22% (92/419) patients overall. The list of all the variants identified are detailed in Supplementary Table S1. PARP-i treatment was commenced in 49 (16%) NELCN women (27 following primary treatment and 22 following recurrence).

*BRCA1/BRCA2* germline and somatic PV concordance:

Concordance of *BRCA1/BRCA2* PV identified through germline and tumour testing was explored. This included 232 paired samples with results from NELCN and 116 paired samples with results from Manchester NHS Trust. There were six *BRCA1/BRCA2* PVs that showed discordance between germline and tumour testing, five in the NELCN cases and one from the Manchester cases, comprising 10.3% of all germline PVs. Five of these six *BRCA1/BRCA2* PVs were LGR that were not detected on somatic testing; one (3%) germline mutation (from NELCN cases) was initially reported in the somatic report but not in the germline. This mutation was then subsequently identified in the germline following re-analysis of the germline sample. The inability of routine somatic testing to reliably identify LGRs is an important finding with implications for those developing and/or implementing OC mainstreaming pathways and for those whose pathways currently use a somatic testing first triage mechanism. It is critical that patients with LGRs are not missed both from a cancer treatment perspective as well as for precision prevention in unaffected relatives with a PV identified through cascade testing.

Pathway improvements:

Changes to the NELCN pathway were incorporated over time to improve logistic efficiencies, communication between team members and timely communication of result to the patient. These included: agreement on a standardised format for reports received from genomic laboratories and omitting of reporting class-1 and class-2 variants. This improved interpretability by cancer clinicians and reduced unnecessary distress in patients.

Initially somatic reports were uploaded as supplementary reports to the original histology result but this caused delays in clinician receiving the information and communicating this to the patient. This was addressed by results being directly sent from the genomic laboratory creating to a shared email-box which was accessed by all members of the clinical team. Responsibility for monitoring and ensuring all results were actioned was subsequently undertaken by the lead medical oncologist.

Electronic communication with electronic request forms being sent directly to cellular pathology lead scientist rather than to the lead histopathologist, triggered the laboratory technician to pull the relevant blocks and slides for the attention of the gynaecological histopathologist, minimising the delay between clinician request and sample being sent to the genomic laboratory.

The NELCN has a Bengali speaking ethnic minority population, which varies from 3% to 33% depending on the borough. All patient facing documents were translated into Bengali to improve engagement and communication with Bengali patients and family members as well as improve decision making. Additionally, a Bengali-speaking clinical member of the extended team, acted as an advocate during genetic counselling.

## 4. Discussion

We demonstrate that unselected concomitant/parallel panel germline and somatic testing at OC diagnosis can be implemented within the NHS setting, and delivered by treating cancer clinicians/professionals through a cancer-MDT coordinated approach. Pre-test counselling was undertaken by all members of the cancer MDT team including medical oncologists, surgical oncologists and CNSs. Consistent with other reports of high uptake rates for *BRCA* testing, [23,32,33,34] we showed this high acceptability extends to panel germline and somatic genetic testing too, with an uptake rate of 97%. PV carriers were younger, more likely to have a strong FH of cancer, HGSC histology and a CRS of 3 at histology. PV status was independent ethnicity, stage at diagnosis, timing of surgery or resection status. We undertook genetic testing prospectively for newly diagnosed patients and also for patients undergoing follow-up. Restricting this to prospective implementation of newly diagnosed cases alone (as has been implemented in some centres) would have missed 19 (19/54, 35.1%) germline PVs which were detected in the follow-up patients, thus significantly affecting screening/prevention options for these unaffected family members. A total of 56% of PVs would have been missed by using an FH based approach alone, reconfirming the importance of unselected testing and a mainstreaming approach. This is consistent with reports from others who also showed that around 50% PVs lacked a strong FH of BC or OC [23,33]. The *BRCA* PV prevalence in our NELCN cohort was higher than the Manchester cohort. Some boroughs in North East of London are known to have an Ashkenazi Jewish (AJ) population and the presence of AJ founder mutations in seven NELCN OC cases (Supplementary Table S1) is a contributory factor towards this as *BRCA* PV are commoner in AJ compared to non-AJ general population OC cases [35]. We found seven AJ BRCA founder mutations in the NELCN cohort but none of these patients self-reported Jewish ethnicity at recruitment. These patients may have had mixed parentage or grand-parentage and been unaware of their ethnicity or may have preferred not to report/disclose Jewish ethnicity. Additionally, NELCN includes 122 women who had previously been diagnosed and were alive at the time of commencement of the study. Although short term survival for *BRCA* PV carriers is higher, we did not find the sub-group of 122 women may be enriched for PV.

Our data show that over 1 in 5 (22%) patients have a PV which can affect their treatment, and 1 in 6 have a germline PV which can also affect predictive testing and screening and prevention in unaffected family members. This is consistent with some other reports in the literature [23,33,36,37]. Testing for a panel which includes *RAD51C, RAD51D, BRIP1* is not currently part of the NHS Genomics test directory and therefore not mandatory across the UK. However, it can if implemented identify an additional 13% (7/54) PVs, with a prevalence of 2.3% in OC patients, whose families can benefit from precision prevention. Rust et al. showed a slight increase in PVs detected with additional *RAD51C/RAD51D* testing but this was not completely unselected in their cohort and was undertaken either sequentially or in those with a strong FH [33]. Our data confirm the benefit of amending the UK test directory criteria to offer multi-gene panel testing to all UK women with OC. Our multi-gene germline test includes high- and intermediate risk genes which have already proven clinical utility [38]. A number of commercially available panels are available today which test for many more (30–100) genes. However, it is important that only genes of established clinical utility are tested for. We are against indiscriminate panel testing for genes without established clinical utility [39,40]. In addition to *RAD51C, RAD51D* and *BRIP1* genes, it would be appropriate for an OC panel to also include *PALB2* and Lynch Syndrome genes going forward. *PALB2* has recently been reported as a moderate risk OC gene [41] and Lynch Syndrome (MMR) genes may be found in another 1% OC patients [42,43,44]. Some initial reports suggest that cascade testing rates may be lower following mainstreaming compared to testing in clinical genetics [34]. However, all our patients with PVs are reviewed in clinical genetics teams, who are responsible for facilitating cascade testing. Additionally, cascade testing rates are likely to increase with longer follow up.

As multiple genes get incorporated into OC testing panels, the reported VUS rate will also increase. Our germline panel VUS rate was 6.6% and is comparable to that reported by others [45,46]. VUS reporting and subsequent management can pose challenges for counselling, variant monitoring and onwards risk management. This will become an increasingly important issue with widening of the panel of genes tested for [47]. Risk reducing surgery, chemoprevention, screening or downstream predictive testing for unaffected family members, is not recommended in individuals with a VUS. Our report also highlights the importance of uniform classification and standardised reporting of class 3 variants (VUS) across genetic laboratories, including the description in clinical reports issued. The Cancer Variant Interpretation Group UK (CanVIG-UK) now provides an exemplar of a multidisciplinary network addressing this nationally [30]. This improves interpretability of reports by cancer clinicians. Appropriate pre-test education of patients and providers is necessary to limit the harm that could result from VUS misinterpretation. While not of immediate direct relevance, a proportion of VUS will be reclassified in the future to PVs and then have implications for the patients and relatives. This reclassification rate has been reported as around 9% in a large cohort [48]. In our cohort, a germline mutation *BRCA1* c.442-22_442-13del reported in somatic but missed in initial germline (identified in re-analysis of germline) was initially reported as Class 3 VUS and subsequently a year on from testing, was re-classified as a PV.

Strengths of this study include prospective design and systematic approach to include all patients including those on follow up, as well as the high acceptability and uptake rates demonstrated with our pathway and testing process. The upfront staff training implemented across the pathway and continued support provided along-with broad stakeholder engagement contributed to improved patient experience and satisfaction. The extra efforts undertaken to engage with our ethnic minority Bengali population is another strength. In order to broaden access and informed decision making we translated information sheets into local Bengali language and trained a Bangladeshi oncology team member who was instrumental in engaging them in genetic counselling. Our analysis also demonstrates likely success rates for tumour testing for different types of samples which can be helpful for counselling patients and planning services. Limitations include lack of qualitative data and long term follow up data on patient outcomes. These are being collected.

Mainstreaming models such as ours delivered by the cancer MDT team enables implementation of large-scale genetic testing at cancer diagnosis. This approach too can encompass more than one pre-test counselling session where needed. A total of 22% women needed and received more than one pre-test counselling session in our study. Most other mainstreaming studies do not report on the number of pre-test counselling sessions needed or if multiple were offered. Our clinical nurse specialists favoured utilising more appointments/consultations prior to recruitment. While we did not undertake a formal quantitative assessment of reasons for multiple consultations, colleague feedback indicates these included, some patients needing more time to assimilate information and reflect on it before deciding and/or the need to discuss further with family before decision making; as well as a clinical assessment of not overloading the patient with too much information at the first setting especially if they were struggling with managing decision making and information related to their cancer care at the appointment. The issues of some initially long consultations and time pressures in a busy oncology clinic also contributed to this. Other examples of models used to deliver unselected genetic testing at OC diagnosis include a genetics team embedded in oncology clinics, [25] genetic nurse coordinated model [24] and medical oncology [32] delivered testing.

Validation and implementation of 3 mm cored biopsies from FFPE tumour blocks enabled time- and resource-efficient processing of archived samples. This is particularly suited for archived FFPE tissue (analysis of retrospective cases) and gave a comparable/higher DNA yield than that obtained through slides. Although, we were unable to test 21% of archived tumour samples, undertaking tumour testing at time of diagnosis for future cases will overcome this. Our pathway now incorporates pathology processing/preparation for genetic testing for all cases at the time of routine histopathology analysis of the initial diagnostic or surgical specimen itself. As a large proportion of failed analysis was pre-treatment diagnostic biopsies, we now routinely obtain additional tissue cores for all women suspected of advanced ovarian malignancy at the time of their diagnostic biopsy. This minimises additional pathology laboratory resources needed and is more cost and time efficient. We also provide estimates of failure rates of diagnostic biopsy (~23%), which is relevant for counselling and management of patients planned for neo-adjuvant chemotherapy. NHS Laboratory guidelines suggest the minimum tumour content for NGS somatic/tumour testing referrals should be 20% [13]. However, we showed benefit of undertaking tumour testing even with <20% content in 45% of such cases. Hence, tumour testing should not be held back in cases with low tumour content as it could be successful in almost half these cases, thus identifying additional women who may benefit from PARP-i treatment.

There has been debate whether both germline and somatic testing should be offered to all; whether unselected germline testing should be offered as first line, followed by somatic testing if germline is negative for PV; or whether reflex somatic testing should be done first, reserving germline if a somatic PV is identified. PVs caused by large genomic rearrangements (LGRs) are missed when PCR-based testing alone is used [49,50]. MLPA is a commonly/routinely used technique to detect LGRs and is found to be highly sensitive and inexpensive [51,52]. LGRs are far more prevalent in *BRCA1* than *BRCA2* genes and have been reported to account for a wide range of *BRCA1* (up to 27%) and *BRCA2* (up to 11%) PVs [53,54,55]. In a large study, LGRs were reported to constitute around 24% of *BRCA1/BRCA2* PVs in high-risk breast/ovarian cancer families, [55] while lower rates are reported in other series and in individuals without strong family histories [53,55,56]. Reports suggest significant ethnic variation in the presence of LGR-related PVs: [55] African (2.4%), Caribbean and Latin American (6.7%), Danish (9.2%) and Spanish ancestry (14.5%) [55,56,57]. A disadvantage of using an initial tumour/somatic testing triage strategy is the possibility of missing LGRs. The 11% LGR-rate in our cohort (6/54) is similar to the LGR rate reported in some high-risk breast and ovarian cancer families [54]. In the majority of diagnostic laboratories, NGS tumour/somatic *BRCA*-testing is not validated for detection of LGRs [50]. While sequential tumour/somatic followed by germline testing may be a less costly approach, [58] this strategy runs the risk of missing some germline PVs, particularly LGRs. This can have significant consequences for cancer prevention in families which are missed. Additionally, although reflex tumour testing can identify PVs seen in the germline, up to 31% of patients found to have a PV in the tumour may not get referred for genetic counselling or germline testing [59]. This highlights a potential limitation of a somatic first strategy, and the need for more robust implementation pathways with built in quality control and fail-safe mechanisms.

In contrast to our findings, a few earlier reports suggest 100% concordance between somatic and germline testing [45,60,61]. However, the proportion of LGRs amongst the *BRCA* mutations reported in these studies is unknown, as these have not been described. It is probable/likely that these studies did not have any LGRs in their mutation spectrum. In our cohort, somatic *BRCA*-testing alone, would have missed 9.2% (4/54) of *BRCA1/BRCA2* germline PVs and seven PVs in *RAD51C/RAD51D* and *BRIP1*, which comprise 20% (11/54) of germline PVs detected from 5-gene panel testing, who can benefit from targeted therapy and downstream predictive testing.

Germline-testing alone would have missed 2% (1/54) germline *BRCA1/BRCA2* PVs, and 15 somatic PVs, comprising 23.1% (16/69) of all *BRCA1/BRCA2* PVs in this cohort, who can benefit from PARP-i treatment. The germline PV missed is an error, which is unlikely to be repeated. A germline first followed by a somatic testing strategy could be an alternative option, but this approach will lead to a longer delay in turn-around times and increase clinician counselling time for giving results as this will need to be done twice. It is also likely to increase the laboratory processing and reporting time and costs, as this is undertaken after initial diagnosis (not contemporaneously with diagnostic reporting). In our experience, a simultaneous or parallel somatic/tumour and germline strategy is a more efficient approach for patients.

## 5. Conclusions

We demonstrate successful implementation of unselected 5-panel germline and concomitant somatic *BRCA1/BRCA2* testing for patients with OC. *BRCA1/BRCA2* germline PVs were identified in 15.5% patients and *BRCA1/BRCA2* somatic PVs in 7.8%. *RAD51C/RAD51D/BRIP1* PVs comprised 13% of PVs and were identified in an additional 2.3% patients. A total of 11% germline PVs are LGRs and are missed by a somatic first testing strategy. A total of 20% of germline PVs would be missed if somatic *BRCA*-testing alone was used to triage for germline testing. A total of 55.6% germline PVs would have been missed by using FH ascertainment alone. The somatic testing failure rate is higher (23%) for patients undergoing diagnostic biopsies. Retrospective archival FFPE tissue testing is feasible using 3 mm punch biopsies from tumour blocks. Our findings favour a prospective parallel somatic and germline panel testing approach as a clinically efficient strategy which maximises variant identification for clinical benefit. The UK Genomics test directory criteria should be expanded to include a panel of OC genes. Formal cost-effectiveness analysis for panel testing is needed and can facilitate wider clinical implementation.

## Figures and Tables

**Table 1:** Factors associated with number of pre-test consultations.

Variation	1 Consultation *n* (%)	>1 Consultation *n* (%)	*p*-Value *
**Member of oncology team undertaking pre-test counselling**			
Medical Oncologist	116/235 (49%)	21/68 (30%)	<0.001
Surgical Oncologist	93/235 (40%)	17/68 (22%)	
Clinical nurse specialist	26/235 (12%)	30/68 (48%)	
**Disease status at the time of counselling**			
New diagnosis of ovarian cancer	127/235 (54%)	40/68 (59%)	0.580
Under follow up	108/235 (46%)	28/68 (41%)	
**Treatment status at the time of counselling**			
Undergoing treatment	155/235 (66%)	50/68 (74%)	0.303
Not on treatment	80/235 (34%)	18/68 (26%)	

* Chi-square test comparing ‘1 consultation and >1 consultation groups’ by variables of type of counselling clinician, disease status and treatment status at time of pre-test counselling.

**Table 2:** Demographic and clinical characteristics NELCN cohort.

Category	No Germline Pathogenic Variants	Germline Pathogenic Variants	Significance	
**Total**	249/303 (82.2%)	54/303 (17.8%)		
**Ethnicity**				
**White**	164/249 (65.9%)	32/54 (59.3%)	*p* = 0.515	
**Black**	23/249 (9.2%)	5/54 (9.3%)		
**South Asian**	39/249 (15.7%)	13/54 (24.1%)		
**Other**	23/249 (9.2%)	4/54 (7.4%)		
**Age in years**				
**Median (IQR)**	61 (51–71)	54 (51–62)	*p* < 0.001	
**Family History**				
**Positive**	28/249 (11.2%)	24/54 (44.4%)	*p* < 0.001	
**Negative**	221/249 (88.8%)	30/54 (55.6%)		
**Histology**				
**HGSC**	207/249 (83.1%)	52/54 (96.3%)	*p* = 0.010	
**All others**	42/249 (16.9%)	2/52 (3.7%)		
**Stage**				
**Early stage**	57/249 (22.9%)	10/54 (18.5%)	*p* = 0.589	
**Advanced stage**	192/ 249(77.1%))	44/54 (81.5%))		
	**No Pathogenic Variants**	**Total Germline or Somatic Pathogenic Variants (PV)**	**Germline PV**	**Somatic PV**
**Total**	234/303 (77.2%)	69/303 (22.8%)*	54/303 (17.8%)	15/232 (6.5%)*
**Timing of surgery**				
**Primary surgery**	115/234 (49.1%)	30/69 (43.5%)	23/54 (42.6%)	7/15 (46.7%)
**Interval surgery**	69/234 (29.5%)	28/69 (40.6%)	23/54 (42.6%)	5/15 (33.3%)
**Delayed surgery**	12/234 (5.1%)	4/69 (5.8%)	2/54 (3.7%)	2/15 (13.3%)
**no surgery**	38/234 (16.1%)	7/69 (10.1%)	1/54 (1.9%)	1/15 (6.7%)
	**significance**	*p* = 0.307		
**Disease status of ovarian cancer at time of counselling**				
**New diagnosis**	126/234 (53.8%)	41/69 (59.4%)	35/54 (64.8%)	6/15 (40%)
**Under follow up**	108/234 (46.2%)	28/69 (40.6%)	19/54 (35.2%)	9/15 (60%)
	**significance**	*p* = 0.463		
**Chemotherapy response score**				
**1**	4/234 (1.7%)	0	0	0
**2**	52/234 (22.2%)	13/69 (18.8%)	12/54 (22.2%)	1/15 (6.7%)
**3**	13/234 (5.6%)	13/69 (18.8%)	9/54 (16.7%)	4/15 (26.7%)
**Not applicable**	165/234 (70.5%)	43/69 (60.0%)	33/54 (61.1%)	10/15 (66.7)
	**significance**	*p* = 0.025		
**Resection (residual disease) status post surgery**				
**R0**	175/234 (74.8%)	54/69 (78.2%)	42/54 (77.8%)	12/15 (80%)
**R1**	14/234 (6.0%)	4/69 (5.8%)	3/54 (5.6%)	1/15 (6.7%)
**R2**	7/234 (3.0%)	5/69 (7.2%)	3/54 (5.6%)	2/15 (13.3%)
**Not applicable**	38/234 (16.2%)	6/69 (8.7%)	6/54 (11.1%)	0/15 (0%)
	**significance**	*p* = 0.276		
**Mutation Prevalence NELCN Cohort**				
	**Gene**	***n***	**Pathogenic (%)**	**VUS (%)**
**NELCN cohort**				
**Germline**	*BRCA1*	303	33 (11%)	3 (1.0%)
	*BRCA2*	303	14 (4.6%)	7 (2.3%)
	*RAD51C*	303	2 (0.7%)	2 (0.7%)
	*RAD51D*	303	3 (1.0%)	2 (0.7%)
	*BRIP1*	303	2 (0.7%)	6 (2.0%)
	Total Germline PVs	303	54 (17.8%)	20 (6.6%)
	Sequence PVs	54	48 (88.9%)	-
	LGR PVs	54	6 (11.1%)	-
**Somatic**	*BRCA1*	232	11 (3.6%)	1 (3%)
	*BRCA2*	232	4 (1.3%)	4 (1.3%)
	Total Somatic PVs	232	15 (6.6%)	5 (2.2%)
**Total PVs**		303	69 (22.8%)	25 (8.3%)

Pathogenic variants = class 4/5 variant in BRCA1, BRCA2, RAD51C, RAD51D, BRIP1. Family history positive = first-degree or second degree relative with ovary and/or breast cancer. HGSC = high grade serous carcinoma. Early stage = stage 1–2; advanced stage = stage 3–4. R0 = zero or nil residual disease, R1 = ≤1 cm residual disease, R2 = >1 cm residual disease. IQR = inter quartile range, PV = Pathogenic variants, VUS = Variants of uncertain significance, LGR- large genomic rearrangements. This table describes outcomes by two groups: (a) with and (b) without germline/somatic pathogenic variants. Two-sided *p*-values were reported for statistical tests comparing these two groups * Results of somatic testing at time of analysis for 71 patients were unavailable (only 232 patients had paired samples). Of these 71 patients 9 had a germline PV.

**Table 3:** Comparison of DNA concentration and yield from FFPE 3 mm core and unstained sections of tumour tissue.

Case ID	DNA Concentration (ng/µL)		DNA Yield (µg)	
	Slides	Punch	Slides	Punch
Case 1	69.35	176.4	6.94	17.64
Case 2	40.16	60.49	4.02	6.05
Case 3	25.12	69.64	2.51	6.96
Case 4	45.19	115.9	4.52	11.59
Case 5	54.02	41.93	5.40	4.19

Table 3 describes the validation data of DNA yield from FFPE 3 mm core biopsies and unstained sections of tumour tissue.

**Table 4:** NELCN tumour tissue *BRCA1/BRCA2* next generation sequencing analysis.

Category	Successfully Reported (*n*,%)	Failed Analysis (*n*,%)
Total number of samples	213/232 (91.8%)	19/232 (8.9%) *
**Type of tissue**		
Pre-chemo diagnostic biopsy	37/48 (77.1%)	11/48 (22.9%)
Primary surgery	104/110 (94.5%)	6/110 (5.5%)
Post-chemo cytoreductive surgery	72/74 (97.3%)	2/74 (2.7%)
**Type of tumour sample**		
3 mm core from FFPE	171/174 (98.3%)	3/174 (1.7%)
5 × 5µM unstained slides	42/58 (72.4%)	16/58 (27.6%)
**Neoplastic content**		
<20%	5/11 (45.5%)	6/11 (54.5%)
20–50%	33/40 (82.5%)	7/40 (17.5%)
>50%	175/181 (96.7%)	6/181 (3.4%)

This table describes the results of BRCA testing of tumour tissue in the NELCN cohort. Results are available for 232 cases. * Of the 19 failed analysis, one had a BRCA1 PV and one a RAD51D PV.

**Table 5:** Mutation Prevalence (Manchester cohort).

	Gene	*n*	Pathogenic (%)	VUS (%)
**Manchester Cohort**				
**Germline**	*BRCA1*	116	8 (6.9%)	1 (0.9%)
	*BRCA2*	116	3 (2.6%)	
	Total Germline PVs		11 (9.5%)	
	Sequence PVs	11	10 (90.9%)	
	LGR PVs	11	1 (9.1%)	
**Somatic**	*BRCA1*	116	7 (6%)	1 (0.9%)
	*BRCA2*	116	5 (4.3%)	1 (0.9%)
	Total Somatic PVs		12 (10.3%)	2 (1.8%)
**Total PVs**		116	23 (19.8%)	

This table describes the prevalence of variants in the Manchester cohort. VUS—variants of uncertain significance. PV—pathogenic variants. LGR—Large genomic rearrangements.

## Data Availability

The data that support the findings of this study are available from the corresponding author, R.M., upon reasonable request.

## Supplementary Materials

The following are available online at https://www.mdpi.com/article/10.3390/cancers13174344/s1, Table S1: List of variants identified through germline and somatic testing.
