# Supplementary material for: Implementation of Multigene Germline and Parallel Somatic Genetic Testing in Epithelial Ovarian Cancer: SIGNPOST Study
Source: Cancers (Basel). 2021 Aug 27;13(17):4344. doi: 10.3390/cancers13174344 (PMC8431198; doi:10.3390/cancers13174344)
Supplement: Supplementary file 1 [file cancers-13-04344-s001.zip › cancers-1312764-supplementary.pdf]

Table S1. Variant description.

Sample ID	Gene mutation	Variant	Variant status	Germline mutation	Somatic mutation
1	BRCA1	c.4065_4068del	pathogenic	yes	yes
7	BRIP1	c.2303_2308del	VUS	yes	no
13	BRCA1	c.1749_1755del	pathogenic	yes	yes
14	BRCA1	c.1749_1755del	pathogenic	yes	yes
16	BRCA2	c.5073dup	pathogenic	yes	yes
17	BRCA1	c.5467G > A	pathogenic	no	yes
23	BRCA2	c.5682C > G	pathogenic	yes	Yes
25	BRCA1	MLPA heterozygous deletion of exon probes 20–23	pathogenic	yes	no
27	BRCA2	c.5423T > C	VUS	VUS	no
28	RAD51C	c.745C > T	VUS	VUS	N/A
29	BRCA2	MLPA Heterozygous deletion exon 21	pathogenic	yes	yes
30	BRCA2	c.5130_5133del	pathogenic	yes	yes
31	BRCA2	c.6953G > A<br>c.7699T > G	VUS	no	yes
33	RAD51D	Heterozygous deletion exon 3	pathogenic	yes	failed analysis
34	BRCA1	c.3083deIG	pathogenic	no	yes
36	BRCA1	c.3454G > A	VUS	yes	N/A
40	BRCA2	c.3868delT	pathogenic	no	yes
47	BRCA1	c.3224delA	pathogenic	no	yes
50	BRCA2	c.6944_6947deITAAA	pathogenic	yes	yes
54	BRCA1	c.3607C > T	pathogenic	no	yes
57	BRCA1	c .1116G > A	pathogenic	no	yes
60	BRCA1	c.68_69del	pathogenic	yes	yes
67	BRCA1	c.4035del	pathogenic	yes	yes
71	BRCA2	c.5159C > A	pathogenic	yes	N/A
73	BRCA1	c.470_471del	pathogenic	yes	yes
77	BRCA2	c.3103G > T heterozygote	pathogenic	yes	N/A
80	BRCA1	c.1204G > T	pathogenic	no	yes
81	BRCA1	c .3607C > T	pathogenic	yes	yes
82	BRCA1	c.68_69del	pathogenic	yes	yes
87	RAD51C	c.93del heterozygote	pathogenic	yes	N/A
87	BRIP1	c.778A > G	VUS	yes	N/A
88	BRCA1	c.3607C > T	pathogenic	yes	yes
90	BRCA1	c.442-22_442-13del	pathogenic	yes	yes
102	BRCA2	c.4436G > C	VUS	yes	yes
102	RAD51D	c.493C > T	VUS	yes	N/A
109	BRCA2	c.4593dup	pathogenic	yes	yes
112	BRCA1	c.5074 + 1G > A	pathogenic	yes	N/A
114	BRCA2	c.3680_3681del	pathogenic	yes	yes
116	BRCA1	c.4068delTCAA	pathogenic	no	yes
121	BRCA2	c.7363dup	pathogenic	yes	yes
128	BRCA1	c.5260G > C	VUS	yes	yes
130	BRCA2	c.45dupT	pathogenic	no	yes
131	BRCA2	c.186delT	pathogenic	yes	yes
134	BRCA1	c.5266dup	pathogenic	yes	yes
135	BRCA2	c.5414A > G	VUS	yes	yes
137	BRCA1	c.5177delG	pathogenic	yes	yes

138	BRCA1	MLPA Heterozygous deletion of exon probes 1–2	pathogenic	yes	no
141	RAD51D	p. (Leu179_Leu182del)	VUS	yes	N/A
141	BRIP 1	c.316C > T	VUS	yes	N/A
142	BRCA1	MLPA Heterozygous deletion of exon 14	pathogenic	yes	no
145	BRCA1	MLPA deletion up-stream exon 1, 2	pathogenic	yes	no
146	BRCA1	c.68_69del	pathogenic	yes	N/A
148	BRCA1	c.3770_3771del	VUS	yes	yes
153	BRIP1	c.778A > G	VUS	yes	no
154	BRCA1	c.3664G > T	pathogenic	yes	yes
160	BRCA1	c.5236C > G	pathogenic	yes	N/A
161	BRIP 1	c.626A > G	pathogenic	yes	no
162	BRCA2	C.7884A > G	VUS	yes	yes
170	BRCA1	c. 4088_4092delinsATAGCA	pathogenic	no	yes
177	RAD51C	MLPA heterozygous deletion in exon probes 4–9	pathogenic	yes	no
178	BRCA2	c.9154C > T	pathogenic	no	yes
184	BRCA1	c.165_166dup	pathogenic	yes	yes
185	BRCA1	c.815_824dup	pathogenic	yes	yes
193	BRCA2	c.6450delA	pathogenic	yes	yes
196	BRCA2	c.5177del,	pathogenic	yes	N/A
202	BRCA2	c.1786G > C	VUS	yes	N/A
203	BRCA1	c.4574_4575del	pathogenic	yes	N/A
205	BRCA1	c.121C > T	pathogenic	no	yes
210	BRCA1	c.1053delA	pathogenic	no	yes
217	BRCA1	c.2405_2406deITG	pathogenic	yes	yes
219	BRCA1	c.5249A > G	VUS	yes	yes
220	BRCA1	c.5512deIG	pathogenic	no	yes
221	BRCA1	c.1823_1826delAGAA	pathogenic	yes	yes
223	BRCA2	c.506A > G	VUS	yes	yes
227	BRIP1	c.791G > A	VUS	yes	no
228	BRCA2	c.8021_8023del	VUS	yes	no
235	BRCA1	c.68_69del	pathogenic	yes	yes
238	BRIP 1	c.3559G > A	VUS	yes	N/A
245	BRCA1	c.5266dup	pathogenic	yes	yes
256	BRCA2	c.1052A > C	VUS	no	yes
258	BRCA1	c.3009_3013deITGAGG	pathogenic	no	yes
263	RAD51D	c.del p.(His250Thrfs*2)	pathogenic	yes	no
267	BRCA1	c.1961dup	pathogenic	yes	yes
272	BRCA2	c.3337G > C	VUS	no	yes
276	BRCA1	c.3841C > G	VUS	no	yes
280	RAD51C	c.175C > G	VUS	yes	yes
281	BRCA2	c.5946delT	pathogenic	yes	yes
286	BRCA1	c.814G > T	pathogenic	yes	yes
288	BRIP1	c.2010dup	pathogenic	yes	no
295	BRCA1	c1749_1755del	pathogenic	yes	yes
298	BRCA1	c.2965_3002del	pathogenic	yes	yes
303	BRCA1	c.80 + 1G > A	pathogenic	yes	failed analysis
20105843	BRCA2	c.6841delG	pathogenic	no	yes
201812844	BRCA1	c.1723G > T	pathogenic	no	yes
20110405	BRCA2	c.2152G > A	VUS	no	yes
18027303	BRCA2	c.755_758del	pathogenic	no	yes

201716703	BRCA1	c.1436_1439delAAAA	pathogenic	no	yes
19021467	BRCA2	p. (Pro3292Ser)	VUS	no	yes
19033012	BRCA1	c.2884G > A	VUS	no	yes
19031554	BRCA1	c.2125_2126insAG	Likely pathogenic	no	yes
19031416	BRCA 1	c.4113deIG	pathogenic	no	yes
18001977	BRCA 2	c.6439_6460del22	pathogenic	no	yes
18016611	BRCA 2	c.7480C > T	pathogenic	no	yes
19019733	BRCA 1	c.9874C > T	pathogenic	no	yes
20108924	BRCA 2	c.1436_1439delAAAA	pathogenic	no	yes
19000765	BRCA 1	c.5309G > T	likely pathogenic	no	yes
19032817	BRCA 1	c.5177G > T	VUS	yes	yes
20106276	BRCA 1	c.4065_4068deITCAA	pathogenic	N/A	yes
19031512	BRCA 2	c.8706A > C	VUS	N/A	yes
19030992	BRCA 1	c.5503C > T	pathogenic	yes	yes
19030926	BRCA 1	c.4065_4068delTCAA	pathogenic	yes	yes
18016940	BRCA 2	c.4631dup	pathogenic	yes	yes
20111102	BRCA 2	c.4478_4481delAAAG	pathogenic	yes	yes
19031824	BRCA 2	c.9382C > T	pathogenic	yes	yes
19000597/8	BRCA1	BRCA1 exon 13 duplication	pathogenic	yes	no
18004131	BRCA1	c.5017_5019delCAC	VUS	yes	yes
20104947	BRCA 1	c.181T > G	pathogenic	yes	yes
20103878	BRCA 1	c.2_27del	pathogenic	yes	yes
20103305	BRCA 1	c.112_113delAA	pathogenic	yes	N/A
20110436	BRCA1	c.2681_2682delAA	pathogenic	yes	yes

VUS—Variant of uncertain significance; Pathogenic—incorporates both pathogenic variants and likely pathogenic variants.
